# Notices

**Published:** 1871

**Authors:** 


Secretary's Office of the Illinois State Medical Society,
T. D. Fitch, M.D., *Sec.*, 296 W. Monroe st.

*To the Members of the Illinois State Medical Society:*

Gentlemen—The Transactions of this Society were nearly ready for distribution, but yet in hands of the publishers at the time of our great fire. The publishers lost everything, and the State Medical Society their transactions for this year. Nothing was saved except a copy of the proceedings which was in our hands and a few of the articles and reports that had been published in the medical journals, and others in pamphlet form by their authors. We shall at once put these into type again, and obtain, if possible, duplicate copies of all the unpublished reports from their authors.

We ask the indulgence of the Society for our seeming tardiness in issuing the Transactions, but many circumstances which seemed beyond our control conspired to delay the issue. We have made direct application to the authors of all unpublished papers, and hope to be able to issue nearly, if not quite, a complete copy within the next thirty days.

In behalf of the Committee on Publication.

T. D. Fitch, M.D.

### *Reunion of the Alumni of the Jefferson Medical College.*

The Alumni Association of the Jefferson Medical College proposes to hold a social reunion during the meeting of the American Medical Association in Philadelphia, in May next. The Alumni of the College are cordially invited to attend.

Those who expect to be present are requested to send their names and addresses to either of the undersigned Secretaries.

J. Ewing Mears, M.D., 222 *S.* 16*th St.*

R. J. Dunglison, M.D., 636 *N.* 18*th St.*

---

### *Prize Essay on "Diseases of Children"—Open for Universal Competition.*

The President of the Medical Society of the county of New York, Dr. Abraham Jacobi, has placed in the hands of its Treasurer four hundred dollars, to be awarded for the best essay on "A History of the Diseases of Infancy and Childhood in the United States, and of their Pathology and Therapeutics."

Competitors will send their essays in English, with motto attached, and the name and address of the writer, with the same motto, in a sealed envelope, to the present Secretary of the Society, Dr. Alfred E. M. Purdy, 123 East Thirty-Eighth street, New York, on or before January 1st, 1873.

The committee are authorized by the society to withhold the prize if the essays submitted should not merit it.

Austin Flint, M.D., Ernst Krackowizer, M.D., Edward S. Dunster, M.D., Committee.

